# Halal cultivated meat: an untapped opportunity

**DOI:** 10.3389/fnut.2023.1196475

**Published:** 2023-07-12

**Authors:** Sophie Attwood, Shahid Jameel, Awal Fuseini, Eman AlKhalawi, Cother Hajat

**Affiliations:** ^1^Real World Health, Oxford, United Kingdom; ^2^Oxford Centre for Islamic Studies and Green Templeton College, Oxford, United Kingdom; ^3^Huddersfield Business School, University of Huddersfield, Huddersfield, United Kingdom; ^4^Department of Family and Community Medicine, King Abdulaziz University, Jeddah, Saudi Arabia; ^5^Public Health Institute, United Arab Emirates University, Abu Dhabi, United Arab Emirates

**Keywords:** alternative protein, plant-based, halal, sustainable diet, cultivated meat and dairy

## Abstract

The global Halal food market is forecast to reach US$1.67 trillion by 2025, growing to meet the dietary demands of a rapidly increasing Muslim population, set to comprise 30% of the global population by mid-century. Meat consumption levels are increasing in many Muslim countries, with important implications for health and environmental sustainability. Alt protein products are currently being manufactured and positioned as one possible solution to reduce the environmental impact of meat consumption, yet, little is currently known about the Halal status of these products, nor the extent to which they appeal to Muslim consumers in emerging markets in Asia and Africa. Here, we explore key considerations regarding the acceptability of alt protein products for Muslim consumers, explore Halal certification requirements in the context of cultivated meat, and examine some unique beliefs within the Islamic faith that may support, as well as impede, widespread adoption of alt protein among the 2.8 billion Muslims of the future.

## Introduction

The role that alternative (‘alt’) proteins, particularly alt meat, will play in transitioning the world’s population to more sustainable eating patterns is hotly debated. Some commentators suggest that investment in alt protein technologies can reduce greenhouse gas (GHG) emissions over 11 times more than other green technology investments (1). However, others are more skeptical about these numbers, and are concerned that the emissions reduction potential of alt protein will be limited by its inability to displace meat and dairy products in peoples’ diets.

Fermented, hybrid or cultivated (i.e., cell-based or cell-cultured) proteins (see Table 1 for definitions and examples) are entirely new concepts to many consumers, and are viewed with hesitation, citing concerns about naturalness, healthiness, compliance with dietary requirements, and overall safety (3). As a result, to benefit climate and biodiversity in a meaningful way, companies that produce and market alt proteins must carefully consider how to address consumer reticence. The overriding goal here is to promote habitual purchasing, beyond initial hype, and encourage direct substitution of animal-based products (4). This will be a complex task given that our diets have otherwise been shaped over millennia by a huge range of factors, including local culture, climate, agricultural practices, the food industry, trade, social relations and belief systems.

**Table 1:** Definitions and examples of different types of alternative protein, reproduced from Hajat and Parkin (2).

Name	Brief description	Example
Plant-based	These products use plant-based ingredients, such as beans, peas, lentils, and soy to create meat substitute products.	One market leader is Beyond Meat, which produces burgers and sausages from pea, mung and fava beans and brown rice. Beetroot and apple extract are added to mimic the coloring of meat.
Cultivated meat	*In vitro* laboratory grown meat from cultured cells. Also known as cell-cultivated, cell-based or cell-cultured meat.	The first ever commercial sale of cultivated meat was by an American start-up, Eat Just, in 2013, served in a restaurant in Singapore. Whilst still not widely available in many regions, there are rapid developments in this industry. Companies producing these products include Memphis Meat and Mosa Meats Inc.
Fermentation-derived processes	Traditional fermentation uses microbial anaerobic digestion to improve the taste or functionality of plant-based ingredients. Biomass fermentation involve the rapid growth of microorganisms which form the basis of the product Precision fermentation uses microorganisms as hosts to produce specific ingredients used in alt-meat.	Soybeans are fermented into tempeh. Mycoprotien Quorn is made from fermenting fungal spores. The heme protein (soy leghemoglobin) added to the Impossible Food burger to improve its distinctive meaty flavor.
Hybrid products	Produced by combining reduced portions of animal-based meat, or cultivated ingredients (i.e., fat) with plant-based ingredients.	The Rebel Meat ‘semi- vegetarian’ burger contains 50% beef and 50% plant-based ingredients.
Insects	Processed edible insects, consumed either whole or ground, and used as ingredients to enhance the nutrient profile of existing products.	Fazer, a Finnish company that manufactures bread using cricket flour, and Eat Grub produce energy bars enriched with cricket powder.
Algae	Processed edible algae, mainly used as food ingredients or dietary supplements.	Examples include Spirulina or Chlorella.

Here, we explore religious belief as one highly influential, yet often overlooked factor that influences the food choices of billions worldwide. At present, around 85% of the global population identify with a particular religion (5), many of which issue clear guidance on the types of food considered acceptable to eat. Examples include the vegetarian diet recommended for Hindus, Buddhists and Jains, Islam’s guidance on Halal and Haram foods, and the Jewish Kosher diet, amongst others. Despite such ubiquity, the role that religious beliefs play in shaping food choices is often neglected by companies developing and manufacturing novel foods. This represents a considerable missed opportunity, especially in the context of the Islamic faith, as we explore below.

## The Muslim market for meat

The global Muslim population is predicted to grow to 2.8 billion people by mid-century, comprising around 30% of the world’s population, with most of this expansion occurring in Asia and Africa (6). By then, it is estimated that India, a Hindu majority country, will be home to an estimated 310 million followers, the most of any nation in the world (6). By contrast, most investment and research into alt protein has so far occurred in the United States (US), which is a majority Christian country with a secular innovation ecosystem, followed by Israel, which is primarily Jewish (7).

**Figure 1:**
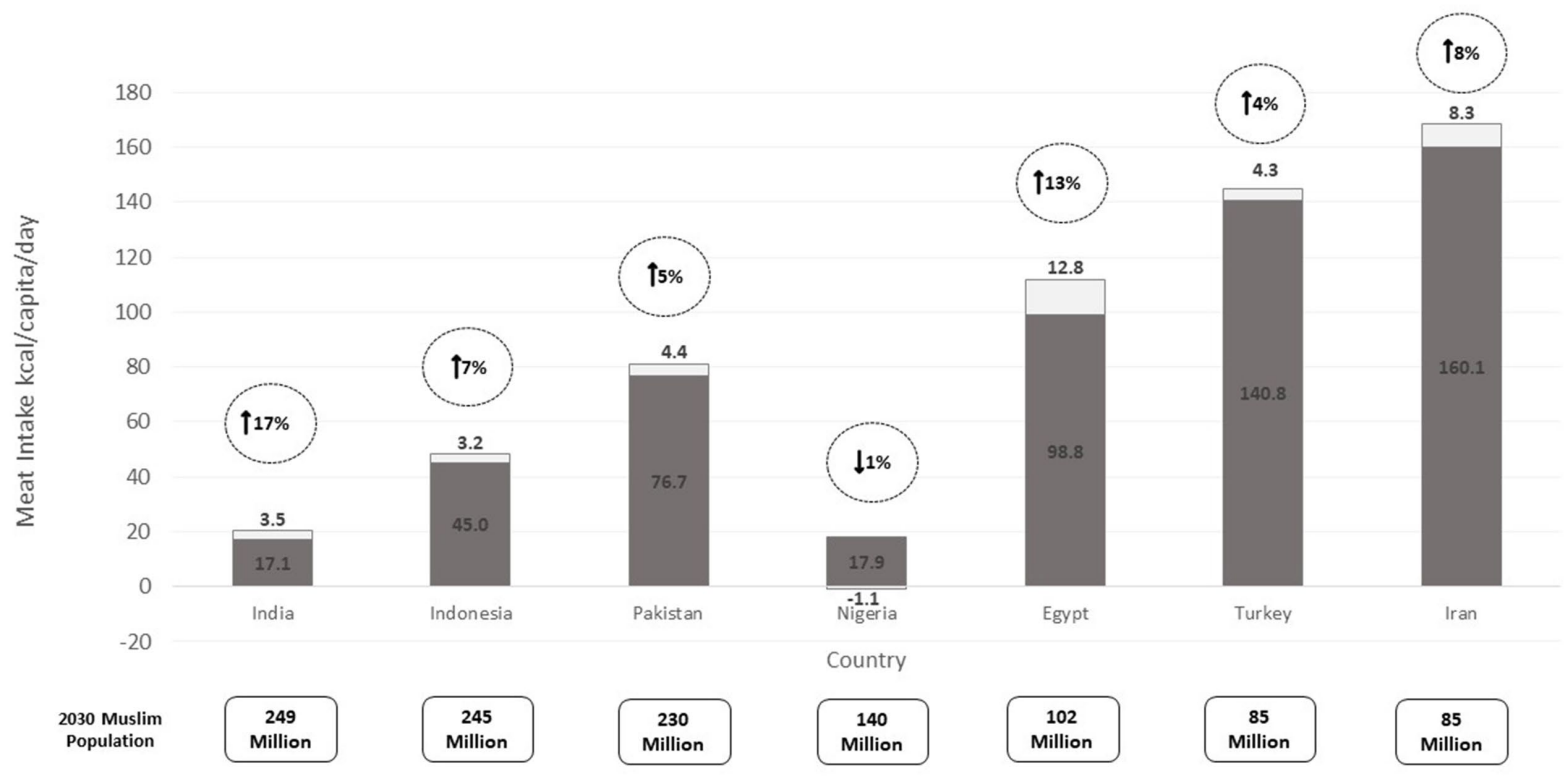
Projected change in meat intake in the most populous Muslim countries by 2030. Data from the OECD FAO Agricultural Output 2022–2023 (9); estimated percentage (%) increase in kcal/capita/day of beef, veal, sheep and poultry meat for the entire population between 2023 and 2030. Non-halal pig meat excluded as default. Population estimates for different religious group sin 2030 provide by Pew Research Centre (6) To note, no baseline data is currently available to estimate changes in meat intake in Bangladesh, Afghanistan, and Iraq – predicted to be home to the world’s 4th, 9th, and 10th largest Muslim populations by 2030.

The projected increase in the world’s Muslim population in Asia and Africa will likely coincide with economic transitions in these regions. Rising income levels tend to lead to dietary changes as more people consume higher value foods, usually those rich in animal protein (8). This is demonstrated in Figure 1, which displays the countries that are estimated to be home to the largest Muslim populations by 2030, and projected changes in meat consumption (i.e., sheep, beef, veal and poultry, combined), from baseline year 2023 (9). Relative increases in meat intake range from 3 to 17%, are highest in India (noting the lower baseline meat consumption level in this country), with only Nigeria registering a decrease from 2023 consumption levels.

## The halal market and dietary requirements

Adherents to Islam are asked to follow a ‘Halal’ diet, which refers to consumption of food and drink consistent with Islamic dietary laws. Box 1, below, outlines Halal requirements, with meat a major focus area. This guidance states that animals must be slaughtered in a prescribed way, and certain types of meat and by-products – including pork and blood products – eschewed. To support Muslims to follow a Halal diet, acceptable foods are certified by different Islamic bodies around the world. These organizations work to ensure that all aspects of production meet necessary requirements, additives and preservatives are Halal compliant, and that meat products have been derived from permissible animals that are slaughtered according to Islamic guidelines (10).

The global Halal food market was valued at US$ 1.27 trillion in 2021 and is forecast to grow, reaching US$ 1.67 trillion by 2025 (for context, global Kosher market revenue was under US$ 20 billion in 2021) (11, 12). In 2020, the trading bloc known as the Organization of Islamic Cooperation (OIC) reported a Halal food export deficit of around US$ 67 billion, indicating strong reliance on imports into major Muslim markets. The greatest volume of this trade comes from non-Muslim producer countries Brazil, India, the US and Russia. Here, technologies that ensure traceability of imported products are essential for consumer trust, and innovation in this area is growing. Most recently, this has included blockchain-enabled platforms to track the origin of Halal meat, and DNA testing kits to ensure imported meat has not been tainted with forbidden animal-based products (13).

Trade imbalance represents a potential food security risk for many Halal markets. This is particularly true for Muslim-majority countries in the Gulf region which, due to climate and terrain, have limited capacity for in-country livestock farming and agriculture to meet national demand, and currently import over 85% of their food and around 62% of their meat (14). As such, alt proteins are not only being considered as potential solutions to climate change, but, if locally produced, can also support national food security goals alongside other innovations like vertical, urban and seawater farming, genetically modified crops and precision agriculture (15).

**BOX 1: Halal Dietary Guidelines.**

- Meat from animals that are permissible and slaughtered in the prescribed way by mentioning the name of Allah (and no other gods or persons)
- Free from any part (or substance) taken or extracted from animals forbidden to be consumed by Muslims.
- Free from any substance declared as unclean according to Islamic law (including products considered to be contaminated with filth*)
- Prepared, processed, produced, or manufactured using utensils, equipment, and/or machinery which are free from any substance declared as unclean according to Islamic law.
- Not contaminated by any food or materials during preparation, processing or storage that do not fulfil the above requirements.
- Plants – all types of plants and their products or derivatives are Halal and can be eaten except if poisonous, intoxicating, and harmful to human health.
- Drinks – all forms of water are Halal, except if poisonous, contain Haram materials, are intoxicating or are harmful to human health. Alcoholic and intoxicating drinks are forbidden. Water mixed with filthy liquids or food laced with wine and alcohol are also not permissible.
- Arising from the idea of ‘clean’ is the concept of ‘tayyib’, which refers to good, pleasant, wholesome, agreeable and delicious, as opposed to ‘khabith’, which refers to impure, harmful and disgusting. Upholding the requirement of ‘tayyib’ in the processing of food includes maximizing hygiene and minimizing contamination.

*Filth, according to Islamic law, includes substances such as pork, blood and carcasses (carrion) which are filth by themselves and cannot be accepted as clean. It also includes otherwise clean substances that are contaminated by filth Adapted from Amabli et al. (16).

## Addressing ethical and religious concerns

If alt protein companies are to succeed in attracting a substantial share of the global Muslim meat market, a fundamental question is whether different types of alt protein can even be certified as Halal. Whilst generally unproblematic from the perspective of 100% plant-based meat analogs, cultivated and hybrid products contain cells derived directly from animals. As yet, no clear guidance has been issued by any Halal certification body regarding the status of these products.

When attempting to address this point, scholars have considered various perspectives; for example, some have argued that cultivated meat contravenes Islam’s ‘Natural Law’, as the production process can be seen as ‘playing God’ (17). Further complications arise from the fact that cultivated meat was originally intended to be made from cells harvested from live animals, rather than those slaughtered. This implies that the resulting cultivated product may not be Halal, unless cells were extracted from a permissible animal slaughtered according to Islamic guidance (17). Indeed, this is one of six key principles that a recent review on the topic suggests must be met in order that cultivated meat can be considered Halal, and is arguably one of the most important hurdles to overcome if cultivated meat products are deemed acceptable for Muslims to eat [see Kashim et al. (18) for an in-depth perspective on this issue]. In his book on animal welfare in Islam, Masri reported that extracting parts of live animals to eat was considered a delicacy in pre-Islamic Arabia, but the practice was subsequently outlawed by the Prophet Mohammed (PBUH) (19).

Another issue of concern for Muslim consumers is the use of cell culture media that are inconsistent with Halal laws. For instance, in many cases, the media in which extracted cells are developed contains fetal bovine serum, which is post-clotted blood fluid obtained from unborn cattle. Blood is considered unclean according to Islamic scriptures [Quran 5:3], and this point has been specifically highlighted by the Malaysian Halal standard (MS 1500/2019) (20). More recently, however, wholly plant-based media have been developed, helping allay the concerns of Halal consumers regarding fetal bovine serum, as well as addressing the requirements of other consumer segments, such as ethical vegans and vegetarians (21).

Similarly, some of the concerns around the acceptability of cultivated meat for Muslim consumers are shared by devotees of other religions. For Jewish consumers who follow Kosher guidance, the Chief Rabbi in Israel ruled for the first time in January 2023 that cultivated steak could be considered a Kosher product (22). This represents one of the first steps toward cultivated foods receiving widespread Kosher certification in the country, and is pivotal move for Israel, which is already home to 57 alt protein start-ups and has declared food technology a national research priority (7). The extent to which similar conclusions will be arrived at by Islamic religious leaders remains to be determined. In 2022, the Assembly of Muslim Jurists of America deemed cultivated meat provisionally permissible by default, provided that the above-mentioned Halal criteria are met. However, an ultimate ruling on the issue will depend on how the technology develops in relation to the source of used stem-cells, additives and the broader health impacts of these products (23). We note that investment in these technologies is also a priority for Muslim competitive markets in the Middle East, particularly the United Arab Emirates (UAE) (24). Abu Dhabi launched their Xprize ‘Feed the Next Billion’ initiative to specifically fund research into development of alt proteins the country (25), and the Middle East’s first plant-based meat factory recently opened in Dubai (26).

## Other factors influencing alt protein adoption in Muslim markets

If and when the issue of Halal certification is resolved, the question of how to boost the appeal of alt proteins to Muslim consumers still remains. Relatively little research exploring Muslim consumers’ perceptions of alt protein is currently available, although the data that does exist suggests potentially greater willingness to try these products compared to non-Muslim consumers (27). For example, one recent study comparing the preferences of British Muslim and Non-Muslim consumers found significantly greater willingness to purchase cultivated meat amongst Muslim consumers, and greater willingness to pay extra for these novel products (28).

Muslims consumers otherwise share many of the same perceived barriers and facilitators to eating alt protein as are observed in other consumer groups across a wide range of countries (28). For example, where research has been conducted, acceptance of novel proteins, particularly cultivated meat, tends to be higher when consumers are more familiar with these products, have lower food neophobia scores, when they taste better, are more affordable, and when consumers are informed of their potential health and environmental benefits compared to traditional meat and dairy (3, 28).

Additionally, there is a range of more specific factors unique to Muslim consumers that are relevant to consider. For example, given that plant-based, hybrid and cultivated products can all be produced in highly controlled environments, this may limit the potential for contamination with non-Halal animal ingredients during production, thereby overcoming fears around product impurity. This is especially likely for cultivated products if the manufacturing process is reviewed and certified by a credible Halal authority that Muslim consumers already know and trust (29).

Availability of plant-based meat alternatives may also circumvent the issue of whether meat should be stunned prior to slaughter, which is generally considered more humane, but some believe is inconsistent with Halal rules (30, 31). Although Islam emphasizes the importance of animal welfare before and throughout the slaughter process, modern farming practices may fail to maintain these principles. As a result, consumers looking for Halal products that ensure animal welfare may be left with few options. More broadly, other qualitative research has revealed that some Muslim consumers recognize additional potential benefits of cultivated meat for the Halal economy, both in terms creating new jobs for halal meat scientists, as well as helping to grow Muslim-owned food businesses (29). Greater adoption of cultivated meat may also be viewed by some Islamic jurists and Halal consumers as a step toward Khilafa (guardianship of nature)[Quran 10:14], which is an important principle related to environmental sustainability. Here, Islamic law states that any new rulings must align with the objective of attaining welfare and warding off harm. As such, the adoption of a diet with a lower environmental impact, *via* consumption of alt protein products, may be considered a way to uphold at least two of the five key principles: the preservation of life and linage.

We also note various other well-known Islamic teachings with implications for health and diet, which may also support movement away from excess meat consumption in Muslim populations. These include the recommendation to avoid wasting food (i.e., *“Eat and drink and do not waste, for God does not love the wasteful.*”[Quran 7:31]), and the Hadeeth (Prophet’s saying) to moderate intake (i.e., “*No child of Adam fills a container worse than his stomach. A few morsels that keep his back upright are sufficient for him. If he has to, then he should keep one-third for food, one-third for drink and one third for his breathing*.” [Jami` at-Tirmidhi (2380), Volume 37, Hadith 77]). In addition, regular fasting is also encouraged, as is taking care of one’s body, as per the saying of the Prophet in response to one of his companions fasting daily *“… your body has a right over you”* [Sahih Al-Bukhari (1977), Volume 30, Hadith 198].

Academic research has proven that religiosity can play a significant role in promoting behavior change, including pro-environmental actions. For example, a recent study by Hassan et al. found that Muslim diners were keen to avoid wasting food to adhere to teachings within the Quran (32). As such, influential Muslims, including Islamic religious leaders, have potential to play an extremely important role in encouraging sustainable and healthy behavioral change, and should be included as key stakeholders in the sustainable diets movement in any Muslim majority country where this is a priority national agenda item (33, 34).

## Conclusion

Muslim consumers’ concerns regarding alt protein are currently poorly understood and rarely addressed by manufacturers of alt protein products. This is despite tremendous potential for market adoption, given the rapid growth and dietary transition occurring in many Muslim populations worldwide. In this piece, we outline key questions that require answering before widespread adoption of alt proteins is likely in Muslim countries. We also recommend further research to address religion-specific barriers to uptake. As decisions made by the Chief Rabbi in Israel attest, religious organizations and leaders can play a vital role in clarifying faith-related concerns about novel foods, helping to encourage vast numbers of followers to adopt more sustainable diets while remaining adherent to the core tenets of their belief systems.

## Data availability statement

The original contributions presented in the study are included in the article/supplementary material, further inquiries can be directed to the corresponding author.

## Author contributions

SA and CH conceived of the article idea. SA contributed as lead authorship. SJ, EA, AF, and CH contributed to writing the final perspective piece. SA edited and formatted the manuscript for submission. All authors contributed to the article and approved the submitted version.

## Conflict of interest

AF works as Halal Sector Senior Manager at the Agriculture and Horticulture Development Board (AHDB) (UK).

The remaining authors declare that the research was conducted in the absence of any commercial or financial relationships that could be construed as a potential conflict of interest.

## Publisher’s note

All claims expressed in this article are solely those of the authors and do not necessarily represent those of their affiliated organizations, or those of the publisher, the editors and the reviewers. Any product that may be evaluated in this article, or claim that may be made by its manufacturer, is not guaranteed or endorsed by the publisher.

## Author disclaimer

The views expressed in this article are the author’s own and do not reflect the views of AHDB.
